# Evaluating the Effects of Metalinguistic and Working Memory Training on Reading Fluency in Chinese and English: A Randomized Controlled Trial

**DOI:** 10.3389/fpsyg.2018.02510

**Published:** 2018-12-12

**Authors:** Tik-Sze Carrey Siu, Catherine McBride, Chi-Shing Tse, Xiuhong Tong, Urs Maurer

**Affiliations:** ^1^Department of Early Childhood Education, The Education University of Hong Kong, Hong Kong, Hong Kong; ^2^Department of Psychology, The Chinese University of Hong Kong, Shatin, China; ^3^Department of Educational Psychology, The Chinese University of Hong Kong, Shatin, China; ^4^Department of Psychology, The Education University of Hong Kong, Hong Kong, Hong Kong

**Keywords:** reading fluency, metalinguistic, working memory, phonological awareness, morphological awareness, reading training, reading intervention, literacy

## Abstract

Children traditionally learn to read Chinese characters by rote, and thus stretching children’s memory span could possibly improve their reading in Chinese. Nevertheless, 85% of Chinese characters are semantic-phonetic compounds that contain probabilistic information about meaning and pronunciation. Hence, enhancing children’s metalinguistic skills might also facilitate reading in Chinese. In the present study, we tested whether training children’s metalinguistic skills or training their working-memory capacity in 8 weeks would produce reading gains, and whether these gains would be similar in Chinese and English. We recruited 35 second graders in Hong Kong and randomly assigned them to a metalinguistic training group (*N* = 13), a working-memory training group (10), or a waitlist control group (12). In the metalinguistic training, children were taught to analyze novel Chinese characters into phonetic and semantic radicals and novel English words into onsets and rimes. In the working-memory training, children were trained to recall increasingly long strings of Cantonese or English syllables in correct or reverse order. All children were tested on phonological skills, verbal working memory, and word reading fluency in Chinese and in English before and after training. Analyses of the pre- and post-test data revealed that only the metalinguistic training group, but not the other two groups, showed significant improvement on phonological skills in Chinese and English. Working-memory span in Chinese and English increased from the pre- to post-test in the working-memory training group relative to other two groups. Despite these domain-specific training effects, the two training groups improved similarly in word reading fluency in Chinese and English compared to the control group. Our findings suggest that increased metalinguistic skills and a larger working-memory span appear equally beneficial to reading fluency, and that these effects are similar in Chinese and English.

## Introduction

Developmental dyslexia is defined as a specific learning disorder in reading and writing despite adequate intelligence, motivation, and educational opportunities (Snowling, 2000). Decades of research have shown that, across a variety of cognitive problems, difficulties in phonological processing are among the core deficits in dyslexia (Ramus, 2003; Ziegler and Goswami, 2005). It follows that reading remediation programs are predominantly concerned with learning to manipulate different phonological units (e.g., Bus and van Ijzendoorn, 1999; Ehri et al., 2001; Kjeldsen et al., 2003; Blachman et al., 2004; Elbro and Peterson, 2004; Hatcher et al., 2004; Suggate, 2016), at least in alphabetic languages. When it comes to learning to read in Chinese, questions arise as to whether such phonology-based training works equally well and whether other methods may also facilitate reading development in this non-alphabetic script.

Several recent studies have directly compared the efficacy of different training methods on improving literacy skills in Chinese (Zhou et al., 2012; Wang and McBride, 2017; Wang et al., 2017). For instance, Zhou et al. (2012) trained Chinese preschool children in phonological awareness, morphological awareness, and homophone awareness, and found that morphological training was more effective in improving their Chinese word reading compared to the other training types. Wang and McBride (2017) contrasted the effects of copying-only training, copying-plus-phonological training, and copying-plus-morphological training on learning to read and write in Chinese among Chinese preschoolers. Greater improvement in Chinese word reading and writing for those who received the copying-plus-morphological training demonstrated the importance of morphological awareness in literacy acquisition in Chinese. Note that these training sessions typically center around analyzing the unique structure and characteristics of Chinese, such as the phonological, morphological, and orthographic information available in Chinese characters/words. Therefore, we group them under the label of metalinguistic training in this paper.

In the present study, we chose to compare the effects of working memory and metalinguistic training on reading fluency in Chinese. We did this for two reasons. First, Chinese is morphosyllabic (DeFrancis, 1984), meaning that characters map onto morphemes and syllables rather than onto phonemes as in alphabetic languages. Hence the alphabetic model of training letter-sound correspondence does not quite apply to Chinese-reading children, particularly those in Hong Kong who do not use phonetic coding systems in learning Chinese (McBride-Chang et al., 2004). Indeed, drill-and-practice is the conventional pedagogy in teaching Chinese characters (Wu et al., 1999). Children in Hong Kong typically learn to read in Chinese via rote memorization due to a lack of a coding system to represent character pronunciations. Also they are subject to massive repetitive copying of characters both at school and at home in Chinese instruction (Lin et al., 2009). Obviously such rote learning demands a heavy working memory load. It is thus unsurprising that children’s working-memory capacity predicts their performance in word reading (Ho et al., 2004; Chung and McBride-Chang, 2011), text comprehension, and writing in Chinese (Guan et al., 2014). Chinese children with reading difficulties also show deficits in working memory (Ho et al., 2004; Peng et al., 2013). Given the relevance of working memory to learning Chinese, a reasonable prediction is that increasing children’s working memory span can enhance their word reading fluency in Chinese.

Second, we targeted metalinguistic training because recent work on Chinese literacy acquisition has taken a more analytic approach to understanding Chinese characters. Therefore we aimed to gather corroborative evidence on whether an intervention that trained children to analyze features of Chinese characters would improve reading fluency. The most common type of Chinese character is the semantic-phonetic compound. It accounts for about 85% of the characters in the Chinese writing system (Zhu, 1988) and for 72% of the Chinese characters that children are expected to learn in primary school (Shu et al., 2003). This type of character contains two basic constituents. While the semantic radical offers a hint about meaning, the phonetic component provides a clue to pronunciation (Ho and Bryant, 1997; Shu and Anderson, 1997). For instance, the Chinese character 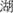 /wu4/ (Cantonese; lake) is made up of the semantic radical 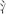, which means water or liquid, and the phonetic component 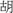 is pronounced /wu4/. It is considered a *fully regular* and *consistent* character – it has the exact same pronunciation as its phonetic part written as a character (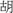/wu4/), and all the compound characters that contain this phonetic radical are pronounced the same (e.g., 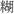/wu4/, 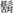/wu4/). However, note that such phonetic information is not entirely reliable. There are *inconsistent* characters in which the compound characters sharing the same phonetics are not pronounced the same way (Yang et al., 2009). For instance, though having the same phonetic radical 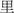/lei5/, the characters 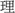/lei5/, 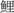/lei5/, 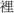/leoi5/, and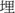/maai4/ are pronounced quite differently in Cantonese. Sometimes the phonetic provides partial information about pronunciation (e.g., 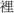/leoi5/ because its phonetic 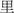/lei5/ has some phonological information in common but not all, e.g., the same onset and tone but different rimes; Shu et al., 2003). In extreme cases the phonetic component offers obscure information about the character pronunciation (e.g., 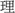/maai4/, so that its phonetic 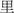/lei5/ is different in all the three phonological elements (i.e., onset, rime, and tone).

Interestingly, despite the probabilistic nature of phonological information in Chinese characters, Chinese children still refer to phonetic components for character pronunciation (Ho and Bryant, 1997; Shu et al., 2000; Anderson et al., 2003). Specifically, they use the pronunciation of the phonetic in reading semantic-phonetic compound characters (e.g., read 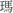/maa5/ by referring to its phonetic radical 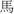/maa5/). When a character contains a bound-phonetics which is unpronounceable (e.g., 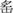), children then infer the character pronunciation by analogy with other characters sharing the same phonetic radical (e.g., 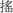/jiu4/, 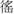/jiu4/, 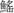/jiu4/; Ho and Ma, 1999; He et al., 2005). Previous intervention studies have demonstrated the effectiveness of using subcharacter information about pronunciation in improving children’s literacy skills in Chinese. In a 5-day intensive training study, Ho and Ma (1999) taught Chinese dyslexic children the structure of compound characters and introduced the functions of phonetic components in regular, semi-regular, and irregular characters. Those who received such explicit phonetic instruction outperformed the control group on Chinese word reading after training. Packard et al. (2006) and Wu et al. (2009) similarly trained Chinese children to identify and analyze the semantic and phonetic components in Chinese characters. The training enhanced children’s performance in reading fluency, vocabulary, reading comprehension, as well as writing Chinese characters from memory.

Anderson et al. (2003) claimed that the partial information about character pronunciation is helpful for assimilating the Chinese characters. We agree with this notion, and see the less-than-perfect clue to character pronunciation as a starting point for children to encode and remember the characters. Beyond this point, however, children still have to learn the correct pronunciations provided by teachers or stated in a dictionary by rote. Hence, in reading such a phonologically opaque script, we argue that both an ability to reflect on the phonetic and semantic components of Chinese words and an ability to hold phonological information in working memory are indispensable resources. One intriguing question that follows is which of these two abilities is more effective in improving reading in Chinese. To answer this question, we designed the current training study to contrast the effects of metalinguistic and working-memory trainings on children’s reading fluency in Chinese.

Notice that our reading test included separate lists of consistent and inconsistent Chinese characters. In this study, we hypothesized training-specific effects on reading different types of characters. We expected that the metalinguistic training would improve children’s learning to read consistent characters as compared to their learning to read inconsistent characters. Phonetic radicals in consistent characters offer reliable cues to character pronunciations. Children might use a more phonological strategy by relying on phonetic radicals when reading consistent characters. Therefore our metalinguistic training with instruction on phonetic radicals would specifically improve learning to read consistent characters. In contrast, phonetic radicals in inconsistent characters are unreliable hints for the correct pronunciation. In learning inconsistent characters children might use rote learning to memorize and recall character pronunciations. Hence, we speculated that children who were trained in working memory might be at an advantage in reading inconsistent characters because they were equipped with an increased working memory capacity to learn the inconsistent character pronunciations.

In addition, we also compared the two training methods in terms of their effects on learning to read in English. English is an alphabetic script which maps onto phonemes as well as other phonological units and, thus, is phonologically more transparent than Chinese. We speculated that learning to analyze word structure in English (i.e., metalinguistic training) is more effective than learning word pronunciations by rote (i.e., working memory training), because phonological information in English words is more reliable in cuing word pronunciations. We also expected that this effect would be more pronounced in learning to read consistent than inconsistent English words.

## Materials and Methods

### Participants

A total of 37 second graders (19 boys; mean age = 7.5 years, *SD* = 0.3) from 30 mainstream primary schools in Hong Kong participated in this training study. All children were native Cantonese speakers with reading skills in the normal range. Parents responded to advertisements posted in online parent-child forums, and were contacted via follow-up phone calls. The children were randomly assigned to three groups. Thirteen children were trained in metalinguistic skills, twelve children were trained in working memory, and twelve children were the waitlist-control group who received training only after the post-test. Two children from the working memory group later dropped out. Parents gave written informed consent on children’s participation at the pre-test. All testing and training sessions were administered individually either at the child’s home or in a laboratory at the university. The children and parents received stationery gifts and HKD300 cash coupon upon completion of the entire training study. The research procedures and written consent form were approved by the Ethics Committee of the Social Science Panel at The Chinese University of Hong Kong.

The group characteristics are listed in Table 1. The three groups did not significantly differ regarding gender, age, non-verbal intelligence, or in Chinese and English oral vocabulary (all *p* > 0.27).

**Table 1:** Group characteristics for training and control groups.

Measure	Metalinguistic		Working memory		Waitlist control	
Gender (male:female)	*6:7*		*4:6*		8:4	
Age (in months) [mean (SD)]	89.23	(3.98)	91.75	(4.61)	89.83	(4.11)
Raven’s Standard Progressive Matrices [mean (SD)]	24.31	(6.01)	27.58	(4.52)	25.83	(4.04)
Chinese vocabulary definition [mean (SD)]	15.00	(8.36)	16.70	(3.50)	15.58	(6.83)
English vocabulary definition [mean (SD)]	6.65	(4.39)	7.55	(4.27)	6.21	(3.33)

### Materials and Procedures

#### Pre-test and Post-test Battery

##### Non-verbal intelligence

At the pre-test we used Raven’s Standard Progressive Matrices (RSPM; Raven et al., 1996) to estimate children’s non-verbal reasoning ability. Children finished Set A to C of RSPM which included 36 black-and-white items. Each item presented a target geometric design with one missing part. The children were instructed to pick the piece that best completed the geometric design among six or eight option patterns. The maximum score of this test was 36.

##### Oral vocabulary

We administered two vocabulary definition tests, one in Chinese and one in English, at the pre-test to measure children’s expressive vocabulary knowledge as a proxy of their general language proficiency in Chinese and in English. The test design was based on the vocabulary subscale of Stanford-Binet Intelligence Scale (Thorndike et al., 1986). The test items and scoring procedure have been used in previous studies on literacy acquisition in Chinese children (e.g., McBride-Chang et al., 2008; Li et al., 2012; Zhou et al., 2012, 2014). The tests consisted of 26 Chinese two-character words and 15 English words that are frequently used in locally published primary school textbooks (Zhuang, 2000). The words were arranged in order of increasing difficulty. For each item, the experimenter read aloud a word and the child was asked to explain the meaning of the word. The answers were scored 0, 1, or 2 based on the accuracy and fullness of the definition given. The test was discontinued when a child failed to define five consecutive words. The maximum score of the Chinese and English tests were 52 and 30, respectively.

We administered the phonological tests, verbal working memory tests, and word reading fluency tests in Chinese and in English at both pre- and post-tests.

##### Word reading fluency in Chinese

We assessed children’s ability to read in Chinese at the pre- and post-tests to evaluate the training effects. In this test, children were presented with two lists of Chinese single-character words separately. The first list included 80 consistent Chinese characters. The consistency value of all consistent characters was greater than 0.8 (mean consistency value^1^ = 0.96). Excluding tone, the consistency value of all the characters in this list was 1, which means characters sharing the same phonetic component sounded the same (e.g., 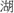/wu4/, 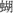/wu4/, 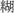/wu4/, 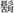/wu4/). The second list had 80 inconsistent Chinese characters (mean consistency value = 0.35), in which characters having a common phonetic component are pronounced quite differently (e.g., 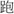/paau2/, 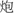/paau3/, 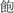/baau2/, 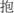/pou5/). Notice that Chinese characters from the two lists were not intermixed. Also, the pre- and post-tests used the same lists of test characters, which are different from those used in the training. Children were given 1 min to read aloud the characters in each list as quickly as possible, skipping over any unknown words. The maximum score for each list was 80.

##### Phonological skills in Chinese

The phonological test included syllable and phoneme deletion to assess children’s abilities to manipulate phonological units in Chinese. This test has been used in several prior studies (e.g., Shu et al., 2008; Cheung et al., 2010; Tong and McBride-Chang, 2010; Li et al., 2012), and the same set of items was used in the pre- and post-test. The syllable-deletion section consisted of two blocks of 10 three-syllable Chinese words: the first block had real words whereas the second block had pseudowords. The experimenter read the words aloud one by one, and the child was asked to delete either the initial, middle, or final syllable. There were 20 one-syllable words in the phoneme-deletion section, with 10 real words and 10 pseudowords. Children were asked to say the syllable that would be left when the initial phoneme was deleted. Testing in each section began with four practice trials with modeling and corrective feedback. The tests stopped if a child made errors in five consecutive items in each section. The maximum score of the test was 40.

##### Verbal working memory in Chinese

We administered a test of non-word repetition to measure children’s verbal working memory in Chinese. Based on previous work which has tested verbal working memory in Chinese children (e.g., Ho et al., 2002, 2004), the test comprised 12 non-word strings which ranged from three to eight Cantonese syllables in length. The syllables were randomly combined so that each non-word string did not carry any lexical meaning. The same set of non-words was used in both the pre- and post-test. In each item, the experimenter read aloud a string of Cantonese syllables and children were asked to repeat the string. One point was awarded for each correct syllable recalled and also for each correct order of consecutive pairs. Two practice trials preceded the test items, and the test was discontinued when a child failed both items in the same span length. The maximum score of this test was 120.

##### Word reading fluency in English

To examine the effect of training on word reading fluency in English, we used the same English word reading test at the pre- and post-tests. This test consisted of two separate lists of English monosyllabic words. The first list contained 80 consistent words for which all other words with the same-spelling rime sound the same (e.g., boy-toy, luck-duck, feet-meet). The second list had 80 inconsistent words for which there exist other words with the same-spelling rime but a different pronunciation (e.g., toe-shoe, five-live, home-come). These test words were different from those used in the training. In each list, children were asked to read aloud the English words in 1 min as quickly and as accurately as possible, and were instructed to skip unknown words. The maximum score for each list was 80.

##### Phonological skills in English

This phonological test assessed children’s abilities to manipulate syllables and phonemes in English. The test items were adapted from those used in previous studies (e.g., Cheung et al., 2010; Li et al., 2012), and the same set of items was used in the pre- and post-test. The syllable-deletion section comprised 20 three-syllable English items, with 10 real words and 10 pseudowords. In each item, the experimenter read aloud an English word and the child was asked to say the word without the initial, middle, or final syllable. The phoneme-deletion section included 20 single-syllable English items, 10 real words and 10 pseudowords. After the experimenter read aloud a word, children were instructed to say the syllable without the initial phoneme. After the four practice items in each section, corrective feedback were given. Testing was discontinued if a child made five consecutive errors in each section. The maximum score of the test was 40.

##### Verbal working memory in English

We tested children’s verbal working memory in English with a non-word repetition task. The test design was modeled on previous work (e.g., Ho et al., 2002, 2004), and the same set of non-words was used in the pre- and post-tests. The test included 12 non-word strings, each with three to eight English syllables. In each item, the experimenter read aloud a string of English non-words. Children were then asked to recall the non-word string in correct order. One point was awarded for each correct non-word recalled and another for each correct order of consecutive pairs. Children became familiarized with the test in two practice items. Testing stopped when children failed both items in the same span length. The maximum score of this test was 120.

#### Training Materials and Protocol

Children in the training groups received 8 weeks of one-to-one tutoring between the pre- and post-tests. The control children received the exact same training (either metalinguistic or working memory) after the post-test. All training sessions involved both parents and undergraduates/postgraduates (named as experimenters thereafter) as tutors. There were four 30-min parent-led and one 1-h experimenter-led tutoring sessions per week. Before training began, tutors completed a 3-h pre-training instruction, given by the first author. The pre-training included an overview of the reading training programs, the structure of Chinese and English languages, and the specific teaching activities and strategies to be followed in the reading training sessions. A log book was given to parents and experimenters to keep a record of the actual training time and the children’s learning performance (i.e., number of words correctly read and written). The first author also met experimenters once a week to obtain individual feedback about children’s learning so as to review and adjust the training protocol.

##### Training stimuli

In the 8-week training, children were taught 96 Chinese and 96 English late-acquired words. Half of the training words were consistent words and half were inconsistent words. Based on a local database of primary school Chinese and English, all the selected words were unfamiliar to second graders in Hong Kong. Pilot study results with 30 second graders also indicated that none of the children could read any of the selected words before training.

##### Metalinguistic training protocol

Parents taught three Chinese and three English words in each tutoring session. The training time between Chinese and English was equally split. The instructions for each Chinese single-character word under the metalinguistic training were as follows:

(1) The child was presented with the target character printed on a training booklet along with its Cantonese pronunciation in jyutping (e.g., 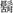 /wu4/).
(2) The parent read aloud the target character and the child followed and repeated the pronunciation.
(3) The parent explained the meaning of the character with the aid of a picture.
(4) The parent showed the child that the target character was composed of a phonetic (e.g., 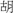; colored in blue) and a semantic component (e.g., 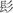; colored in red).
(5) The parent introduced two other characters sharing the same phonetic component as the target character (e.g., 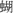/wu4/, 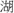/wu4/) to show the child that a phonetic component contained information about the pronunciation of a character.
(6) The parent went on to introduce two other characters sharing the same semantic component as the target character (e.g., 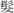, 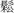) to show the child that a semantic component provided information about the meaning of a character.
(7) The child was asked to write the target character 5 times.

For English words, we taught children to analyze the words into onset and rime. The instructions were as follows:

(1) The child was presented with the target word printed on a training booklet along with its pronunciation transcribed in International Phonetic Alphabet (e.g., trick /trIk/).
(2) The parent read aloud the target word and the child followed and repeated the pronunciation.
(3) The parent explained the meaning of the word with the aid of a picture.
(4) The parent showed the child that the target word was composed of an onset (e.g., tr- /tr/; colored in blue) and a rime (e.g., -ik / Ik/; colored in red).
(5) The parent introduced two other words sharing the same onset as the target word (e.g., try /tra I/, train /tren/).
(6) The parent went on to introduce two other words sharing the same rime as the target word (e.g., kick /k Ik/, pick /p Ik/).
(7) The child was asked to write the target word five times.

Each session ended with reinforcement practice where children were asked to read aloud and write the three Chinese and three English words learned in that session. Parents were instructed to praise correct responses and to correct incorrect responses.

Trained experimenters followed up the training after four parent-led sessions each week. Each experimenter-led session began with a review of all the 12 Chinese and 12 English words learned over the previous week. Children were asked to read aloud and write down the words, then corrective feedback was given by the experimenter. Next, in the Chinese session, children were instructed to name any characters that shared the same phonetic or the same semantic component as the 12 target characters (beyond those presented in the training booklet). If a child failed to recall any, the experimenter guided the child to search for these words in the child’s school textbook. This activity trained the children to note and extract phonetic and semantic information from Chinese characters. Similarly, in the English session, children were guided to name any words that shared the same onset or the same rime as the 12 target words, with the aid of the child’s school textbook.

Children received morphological training in the last 2 weeks. Parents taught two-character Chinese words and English compound words, and asked the child to identify the common morpheme among two to three words. In experimenter-led training, after a review of the words learned, children were asked to name any two-character Chinese words and English compound words that shared the same morpheme as the target words. In addition, they were guided to use the common morphemes to create novel Chinese and English words.

##### Working memory training protocol

The children that received the working memory training were exposed to the exact same Chinese characters and English words as those in the metalinguistic training group. The training activities were as follows:

(1) The child was presented with the target word printed on a training booklet (without any Cantonese or English pronunciation).
(2) The parent read aloud the target word, then the child followed and repeated the pronunciation.
(3) The parent gave the meaning of the word with the aid of a picture.
(4) The child was asked to write the target word five times.
(5) Word span task in Chinese or in English: For the Chinese task, we used the target words to form strings of Chinese words. The span length ranged from two to nine Cantonese syllables. The target words were repeated in a randomized order when the span length was longer than three. In each item the experimenter read aloud a string of Chinese words (i.e., the target words). The child was asked to repeat the string in the correct or reverse order. There were three items in each span length. If the child succeeded in two out of three items in each span length, he/she proceeded to the next span length, otherwise he/she repeated the same span length. This procedure was intended to stretch the children’s working-memory span when they were ready for a longer span length. The English task followed the same procedure, except that English words were used.

Before the session ended, children were also asked to read aloud and write the words learnt. Parents were asked to give corrective feedback on the children’s responses.

In the experimenter-led follow-up training session, children first read aloud and wrote all 12 Chinese and 12 English words learned as a review. The experimenter then introduced two cross-modal word span tasks. In the *oral-to-visual* word span task, the 12 target Chinese or English words were printed (font size = 150) on a large cardboard (60 cm × 40 cm). The experimenter read aloud strings of Cantonese or English syllables (i.e., the target words), and the child was asked to tap the word on the cardboard in correct or reverse order. In the *visual-to-oral* word span task, the experimenter presented strings of target words visually, one by one, on Powerpoint slides. The child was then asked to orally recall the words in a correct or reverse order. Again, the child stayed in the given span length until he/she succeeded in two out of the three items.

### Statistical Analyses

First of all, the training log books indicated that parents from the three groups completed all the designated exercises with their child. The total time spent on the training was also comparable across the three groups, *F*(2,32) = 0.33, *p* > 0.05. Therefore, we can reasonably assume that the training groups were equally compliant with the training.

Next, we computed repeated measures ANOVAs to evaluate the effects of different training methods. The ANOVAs on phonological skills and working memory consisted of the between-subject factor group (metalinguistic vs. working-memory vs. control) and the within subject factors time (T1 pre-test vs. T2 post-test) and language (Chinese vs. English). The ANOVA on reading fluency contained the additional within-subject factor consistency variable (consistent vs. inconsistent characters/words). Follow-up *t*-tests comparing pre- to post-test results were conducted to facilitate interpretation.

## Results

Table 2 summarizes the means and standard deviations of children’s performance on all measures at pre- and post-tests, organized by groups. At the pre-test, the three groups were comparable in their phonological skills [Chinese: *F*(2,34) = 0.134, *p* = 0.875; English: *F*(2,34) = 0.717, *p* = 0.495], verbal working memory [Chinese: *F*(2,34) = 0.076, *p* = 0.927; English: *F*(2,34) = 0.329, *p* = 0.722], and word reading in English [*F*(2,34) = 0.575, *p* = 0.568] before training. The groups tended to differ in word reading in Chinese [*F*(2,34) = 2.658, *p* = 0.085]. We submitted the pre- and post-tests data to repeated-measures ANOVAs and the results are reported below. For Chinese word reading, we added pre-test word reading as a covariate in one of the analyses to test whether post-test results could be explained by pre-test differences (see below).

**Table 2:** Pre-test and post-test mean performances on all measures for training and control groups.

	Metalinguistic		Working memory		Waitlist control	
Measure	*M*	*SD*	*M*	*SD*	*M*	*SD*
**T1 pre-test**						
Chinese phonological skills	23.38	9.84	23.90	9.36	22.92	9.77
Chinese non-word repetition	14.38	5.53	15.00	8.88	15.33	9.32
Chinese word reading fluency – consistent	31.38	14.63	29.30	13.82	45.42	15.38
Chinese word reading fluency – inconsistent	28.46	14.69	25.90	11.00	35.92	14.07
English phonological skills	21.00	8.61	24.70	11.61	19.92	11.94
English non-word repetition	5.85	3.85	5.90	2.64	7.00	4.02
English word reading fluency – consistent	26.31	18.22	29.00	19.79	22.67	19.00
English word reading fluency – inconsistent	26.54	20.35	31.90	22.66	20.08	14.98

**T2 post-test**						
Chinese phonological skills	31.92	8.71	26.00	8.87	23.33	9.37
Chinese non-word repetition	13.69	8.29	23.30	6.36	15.08	8.45
Chinese word reading fluency – consistent	44.31	15.68	38.70	15.94	46.67	14.62
Chinese word reading fluency – inconsistent	35.31	16.31	31.80	12.53	37.50	15.78
English phonological skills	30.38	8.68	26.30	10.06	22.17	9.03
English non-word repetition	7.00	4.36	10.20	4.16	7.50	4.21
English word reading fluency – consistent	34.85	20.44	37.70	23.20	24.42	19.23
English word reading fluency – inconsistent	32.15	20.53	38.40	24.93	21.83	15.87

### Word Reading Fluency

**FIGURE 1:**
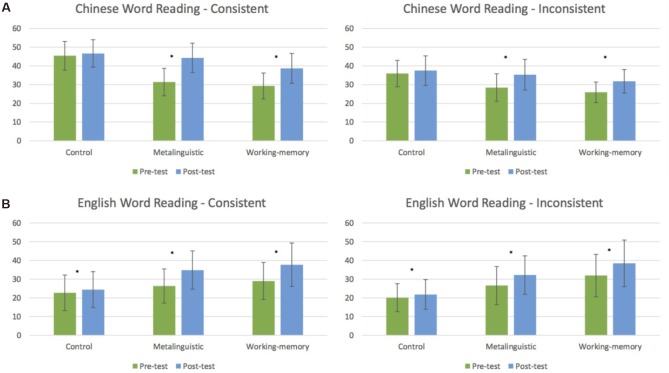
Pre(green)–post(blue) training effects in consistent and inconsistent word reading in Chinese **(A)** and English **(B)** for the three groups (control group, metalinguistic training group, and working memory training group). Error bars represent standard deviations. ^∗^Indicates significant training effects.

Children’s mean performance on word reading fluency in Chinese and English before and after training are shown in Figure 1 for both consistent and inconsistent stimuli. The results of the repeated measures ANOVAs are summarized in Table 3. Accordingly, word reading fluency increased from the pre- to post-test (Time, *p* < 0.001), but more for the two training groups compared to the waitlist control group (Time × Group, *p* < 0.001). This training advantage for the metalinguistic and working memory groups was more pronounced for the consistent than the inconsistent items (Time × Group × Consistency, *p* < 0.05). This three-way interaction also modulated the main effect of consistency (*p* < 0.001) with higher reading fluency for consistent than inconsistent items and its interaction with group (*p* < 0.01). The three-way interaction also modulated a time by consistency interaction reflecting an increase of the consistency effect from the pre- to post-test. In addition, Chinese stimuli were read more fluently than English stimuli (Language, *p* < 0.05), and the consistency effect was larger in Chinese than English (Language × Consistency, *p* < 0.001).

**Table 3:** Results of repeated measures ANOVAs on word reading for combined and separate language analyses.

Effect	Chinese vs. English	Chinese only	English only
T	***F*(1,32) = 125.9, *p* < 0.001,** $\eta_{p}^{2}$ **= 0.80**	***F*(1,32) = 121.8, *p* < 0.001,** $\eta_{p}^{2}$ **= 0.79**	***F*(1,32) = 64.5, *p* < 0.001,** $\eta_{p}^{2}$ **= 0.67**
C	***F*(1,32) = 75.2, *p* < 0.001,** $\eta_{p}^{2}$ **= 0.70**	***F*(1,32) = 65.9, *p* < 0.001,** $\eta_{p}^{2}$ **= 0.67**	*F*(1,32) = 1.1, *p* = 0.299, $\eta_{p}^{2}$ = 0.03
G	*F*(2,32) = 0.0, *p* = 0.986, $\eta_{p}^{2}$ = 0.13	*F*(2,32) = 1.4, *p* = 0.263, $\eta_{p}^{2}$ = 0.08	*F*(2,32) = 1.1, *p* = 0.357, $\eta_{p}^{2}$ = 0.06
L	***F*(1,32) = 4.9, *p* = 0.034,** $\eta_{p}^{2}$ **= 0.13**	n.a.	n.a.
T × G	***F*(2,32) = 17.8, *p* < 0.001,** $\eta_{p}^{2}$ **= 0.53**	***F*(2,32) = 20.8, *p* < 0.001,** $\eta_{p}^{2}$ **= 0.57**	***F*(2,32) = 7.6, *p* = 0.002,** $\eta_{p}^{2}$ **= 0.32**
C × G	***F*(2,32) = 7.9, *p* = 0.002,** $\eta_{p}^{2}$ **= 0.33**	*F*(2,32) = 2.3, *p* = 0.115, $\eta_{p}^{2}$ = 0.13	***F*(2,32) = 3.9, *p* = 0.031,** $\eta_{p}^{2}$ **= 0.20**
T × C	***F*(1,32) = 13.3, *p* < 0.001,** $\eta_{p}^{2}$ **= 0.29**	***F*(1,32) = 11.5, *p* = 0.002,** $\eta_{p}^{2}$ **= 0.26**	***F*(1,32) = 4.2, *p* = 0.049,** $\eta_{p}^{2}$ **= 0.11**
L × G	***F*(2,32) = 3.9, *p* = 0.030,** $\eta_{p}^{2}$ **= 0.20**	n.a.	n.a.
L × C	***F*(1,32) = 25.6, *p* < 0.001,** $\eta_{p}^{2}$ **= 0.44**	n.a.	n.a.
L × T	*F*(1,32) = 1.5, *p* = 0.234, $\eta_{p}^{2}$ = 0.04	n.a.	n.a.
T × C × G	***F*(2,32) = 4.6, *p* = 0.017,** $\eta_{p}^{2}$ **= 0.22**	***F*(2,32) = 4.5, *p* = 0.019,** $\eta_{p}^{2}$ **= 0.22**	*F*(2,32) = 1.2, *p* = 0.324, $\eta_{p}^{2}$ = 0.22
L × T × G	*F*(2,32) = 2.2, *p* = 0.129, $\eta_{p}^{2}$ = 0.12	n.a.	n.a.
L × C × G	*F*(2,32) = 0.4, *p* = 0.701, $\eta_{p}^{2}$ = 0.02	n.a.	n.a.
L × T × C	*F*(1,32) = 1.4, *p* = 0.241, $\eta_{p}^{2}$ = 0.04	n.a.	n.a.
L × T × C × G	*F*(2,32) = 0.8, *p* = 0.444, $\eta_{p}^{2}$ = 0.05	n.a.	n.a.

*L, language; T, time; C, consistency; G, group; n.a, not applicable. Bold print indicates significant effects.*

Given the strong *a priori* assumption that meta-linguistic training would be more effective relative to the working memory training in English compared to Chinese, the lack of a modulation of the training effects by language was surprising. A lack of an interaction with language, however, does not necessarily mean that the group by training interactions occur in both English and Chinese. We therefore computed follow-up repeated measures ANOVAs separately for each language. Pre–post *t*-tests were computed separately for each group and consistency condition, in order to interpret the results of both the overall and the language-specific ANOVAs.

#### Word Reading Fluency in Chinese

The results of the repeated measures ANOVAs for Chinese are listed in Table 3. Reading fluency increased from the pre- to post-test (Time, *p* < 0.001), particularly in the metalinguistic and working-memory groups compared to the waitlist control group (Time × Group, *p* < 0.001). This training advantage of the metalinguistic and working memory groups was more pronounced for the consistent than inconsistent Chinese characters (Time × Group × Consistency, *p* < 0.05). This three-way interaction also modulated the consistency main effect (*p* < 0.001) with higher reading fluency for consistent than inconsistent characters, and also the consistency by time interaction (*p* < 0.01).

*Post hoc t*-tests revealed that both the metalinguistic and working memory training groups increased their reading fluency of consistent [metalinguistic: *t*(12) = −8.34, *p* < 0.001; working memory: *t*(9) = −7.58, *p* < 0.001] and inconsistent [metalinguistic: *t*(12) = −5.52, *p* < 0.001; working memory: *t*(9) = −4.05, *p* < 0.01] characters from before to after training. The pre–post change, however, was not significant for the control children [consistent: *t*(11) = −1.46, *p* = 0.171; inconsistent: *t*(11) = −1.53, *p* = 0.152].

Given the trend toward differences in Chinese word reading before training between the groups, we also computed a repeated measures ANOVA on post-test reading fluency measures with the within-subject factor consistency (consistent vs. inconsistent) and the between subject factor group (metalinguistic vs. working-memory vs. control) and using pre-test reading fluency (average across consistent and inconsistent conditions) as a covariate (Rausch et al., 2003). The results revealed significant main effects of group [*F*(2,31) = 22.46, *p* < 0.001, $\eta_{p}^{2}$ = 0.592] and consistency [*F*(1,31) = 9.17, *p* < 0.01, $\eta_{p}^{2}$ = 0.228] suggesting that post-training group differences in Chinese word reading fluency could not be explained by pre-training group differences.

#### Word Reading Fluency in English

The results of the repeated measures ANOVAs for English are again detailed in Table 3. Reading fluency increased from the pre- to post-test (Time, *p* < 0.001), especially for the metalinguistic and working memory groups compared to the waitlist control group (Time × Group, *p* < 0.01). This training advantage, however, was not significantly larger for consistent than inconsistent words despite a pattern in the means (Time × Group × Consistency, *p* = 0.32). Similar to Chinese, the consistency effect with higher reading fluency for consistent than inconsistent words increased from the pre- to post-test (Time × Consistency, *p* < 0.05). In addition, consistent words were read more fluently in the metalinguistic and working-memory groups, whereas the control group showed a slightly reversed pattern (Consistency × Group, *p* < 0.05).

*Post hoc t*-tests revealed that reading fluency increased from the pre- to post-test in both metalinguistic and working memory groups for both consistent [metalinguistic group: *t*(12) = −5.72, *p* < 0.001; working memory group: *t*(9) = −5.10, *p* < 0.001] and inconsistent items [metalinguistic group: *t*(12) =-3.77, *p* < 0.01; working memory group: *t*(9) = 3.32, *p* < 0.01]. Although the pre–post increases were smaller in the control group, they were still significant [consistent: *t*(11) = −2.51, *p* < 0.005; inconsistent: *t*(11) = −2.78, *p* < 0.05].

### Phonological Skills

**FIGURE 2:**
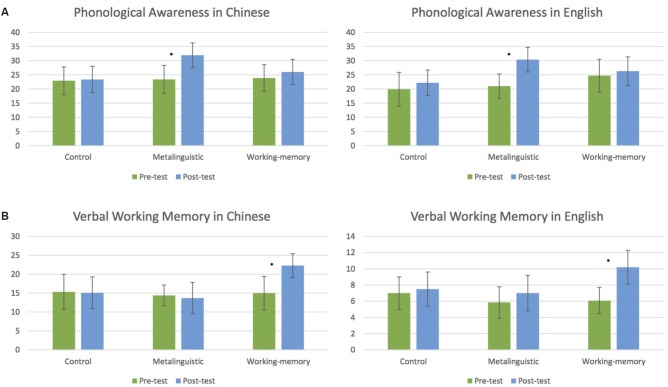
Pre(green)–post(blue) training effects in Chinese and English for phonological awareness **(A)** and verbal working memory **(B)** for the three groups (control group, metalinguistic training group, and working memory training group). Error bars represent standard deviations. ^∗^Indicates significant training effects.

Children’s mean performance on the Chinese and English phonological tests at pre- and post-tests are presented in Figure 2. The repeated measures ANOVA revealed that performance increased from the pre- to post-test [Time, *F*(1,32) = 18.80, *p* < 0.001, $\eta_{p}^{2}$ = 0.370], particularly for the metalinguistic group [Time × Group, *F*(2,32) = 7.43, *p* < 0.01, $\eta_{p}^{2}$ = 0.317]. No other effects were significant (all *p*s > 0.14).

*Post hoc t*-tests revealed that phonological scores increased from the pre- to post-test in the metalinguistic group for both Chinese [*t*(12) = −4.46, *p* < 0.001] and English [*t*(12) = −4.22, *p* < 0.01], but no significant increase was found for the other two groups in either of the two languages (all *p*s > 0.21).

### Verbal Working Memory

Figure 2 also presents children’s mean performance on Chinese and English non-word repetition tasks at both time points. The repeated measures ANOVA revealed that working-memory performance increased from the pre- to post-test [Time, *F*(1,32) = 12.74, *p* < 0.01, $\eta_{p}^{2}$ = 0.285], particularly for the working-memory group [Time × Group, *F*(2,32) = 10.02, *p* < 0.001, $\eta_{p}^{2}$ = 0.385]. In addition, performance was better for Chinese than for English stimuli [Language, *F*(1,32) = 48.19, *p* < 0.001, $\eta_{p}^{2}$ = 0.601]. No other effects reached significance (*p*s > 0.15).

*Post hoc t*-tests revealed that working-memory performance increased from the pre- to post-test in the working memory group in both Chinese [*t*(9) = −2.30, *p* < 0.05] and English [*t*(12) = −2.91, *p* < 0.05], but that the pre–post changes were not significant in the other two groups in neither of the two languages (*p*s > 0.37).

## Discussion

The present study aimed to evaluate two types of reading interventions to improve reading fluency in Chinese and English. Children were either taught to analyze the structure of Chinese and English words (i.e., identify information about pronunciation and meaning in Chinese characters and identify onset and rime in English words; identify morphemes in compound words), or trained to memorize increasingly long strings of Chinese and English syllables. The results indicated that both metalinguistic and working memory training could effectively enhance the respective skills they were meant to train within each language. Despite the training-specific effects, the two training groups improved more in Chinese reading fluency than the control group and this training effect was more pronounced for consistent characters. In other words, a better ability to analyze Chinese characters seems as beneficial to reading fluency in Chinese as an increased working memory capacity. Moreover, these beneficial effects seem not to be fundamentally different for reading Chinese and English, as they could also be found for the metalinguistic and working memory training in English.

### Training Effects on Reading Fluency in Chinese

Our findings contribute to the literature on literacy intervention in Chinese in several ways. First, our study joins prior studies in demonstrating the causal influences of phonological (syllable awareness in particular) and morphological training on learning to read in Chinese (e.g., Chow et al., 2005, 2008; Packard et al., 2006; Wu et al., 2009; Zhou et al., 2012, 2015; Wang and McBride, 2017). Indeed, with our metalinguistic training that combined phonological and semantic training, children learned to analyze character and word structure in Chinese for pronunciation and meaning. Thus our results support the combined effects of phonological and semantic knowledge of Chinese characters in learning to read aloud in Chinese (Zhou et al., 2015).

Second, we evaluated if training in verbal working memory could be translated into reading gains in Chinese. This attempt is an important addition to the literature. Recent meta-analyses have cast doubt on the effectiveness of working-memory training in conferring benefits on general cognitive and scholastic performance (Melby-Lervåg and Hulme, 2013; Melby-Lervåg et al., 2016; though see Klingberg, 2010; Au et al., 2015, 2016, for the counter-argument). Despite this, our data indicate that working-memory training gives both near- and far-transfer effects. Compared to waitlist controls, children who were trained with word span tasks for 8 weeks had a larger verbal working memory span in Chinese immediately after training. These children also improved on their ability to read Chinese characters after training. Our findings, together with other emerging evidence from working-memory training studies in Chinese (Luo et al., 2013; Yang et al., 2017), suggest that working-memory training is effective in facilitating reading aloud in Chinese.

Third, our study showed that the reading training which included consistent and inconsistent characters led to a larger increase in reading fluency for consistent characters. The larger increase for consistent characters, however, was not restricted to the metalinguistic group and also occurred in the working-memory group which did the working-memory training with the same stimulus material. Thus, mere exposure to consistent and inconsistent characters during training may be sufficient to sensitize children to consistency properties and enable them to draw on this information for reading.

Crucially, our study enriches the literature by contrasting the effects of two different teaching approaches to reading acquisition in Chinese. There has been separate evidence that metalinguistic and working-memory training can each improve reading skills in Chinese (e.g., Packard et al., 2006; Wu et al., 2009; Zhou et al., 2012; Luo et al., 2013; Wang and McBride, 2017; Yang et al., 2017). However, due to the complex and ambiguous phonological information in the Chinese script, we wondered if analyzing character and word structure in Chinese or learning character pronunciation by rote might be more promising in learning to read in Chinese. Hence we directly compared their training effects in a single study. We found domain-specific training effects that the metalinguistic and working memory training could enhance the respective skills that they intended to train. To our surprise, our data showed that an increased ability to reflect on character and word structure in Chinese is as beneficial as a larger working memory capacity in improving reading fluency in Chinese. Though the training produced similar reading gains at the behavioral level, the mechanisms underlying the improvements remain unknown (see “directions for future research” below).

### Training Effects on Reading Fluency in English

The training effects in English were similar to those in Chinese. Although the control children also improved in their reading skills in English from the pre- to post-test, this increase was larger for the metalinguistic and the working-memory groups. The increase in phonological skills and in reading fluency in the children trained in metalinguistic skills is in agreement with a large number of studies in English and in other alphabetic languages (e.g., Bus and van Ijzendoorn, 1999; Ehri et al., 2001). More surprising is the effect found in the working-memory training group. As in Chinese, the working-memory group specifically increased in working-memory performance, but also showed an improvement in reading fluency, similar to the metalinguistic group. We speculate that such a beneficial effect on reading fluency in English may have to do with the use of linguistic material in our working-memory training. From the meta-analysis by Melby-Lervåg and Hulme (2013), we found that the studies that failed to find a large effect size on word decoding or vocabulary mostly used non-linguistic materials such as digits, shapes, pictures and sounds of objects as training stimuli (e.g., Horowitz-Kraus and Breznitz, 2009; Holmes et al., 2010; Van der Molen et al., 2010; Shiran and Breznitz, 2011). Interestingly, when word endings and complete words were used in working memory training, a significant large effect on children’s vocabulary was reported (Alloway and Alloway, 2009). This suggests that working memory training may also have beneficial effects on literacy acquisition in English, at least when linguistic material is used as in the current study. Whether such beneficial effects of working-memory training are restricted to learning English as a second language, or even more restricted to learning English as a second language in native Chinese speakers remains to be shown in future studies.

### Limitations and Directions for Future Research

A limitation of the current study is the small group size with 13 and 12 children in the metalinguistic and control groups, respectively, and only 10 children in the working-memory group. Though we randomly assigned the children into three groups, the small group size may contribute to slight pre-existing group differences before training. For instance, the control group performed better than the two other groups in Chinese word reading at the pre-test, though the difference is not statistically significant. Considering all the abilities tested at the pre-test, we think the three groups are reasonably comparable. Yet having a larger group size can further ensure more equivalent groups.

We should also be cautious in interpreting the results because our control group was a waitlist control who did not engage in comparable activity between the pre- and post-test. Without an active control group, some may speculate that the effects found in the metalinguistic and working memory groups were driven by the one-to-one attention given by the parents or experimenters during training. Having said that, the domain-specific effects with working memory gains in the working memory group and phonological gains in the metalinguistic group argue against this notion. The domain-specific effects in the two training groups show that our interventions indeed worked and lend the results credibility.

Another potential limitation of the study is that training was partly provided by parents who may have conducted the training less reliably. However, the training log books showed that all parents completed all the designed training activities with their child. The parents across the three groups also spent comparable time on the training. Moreover, we had trained experimenters who visited the children once per week to monitor the progress and quality of the parent training. We believe that these weekly experimenter training could reduce differences in training quality and increase reliability of the parent training.

Even though the current study did not find differences between metalinguistic and working memory training, such effects may exist but could have gone undetected in the current study for several reasons: First, the small group size may not have provided sufficient power to detect smaller effects between the training types. Second, the two drop-outs were both from the working memory training group which suggests that the effect of the working memory training may be overestimated. Third, our participants were second-grade children who were beginning readers. Their limited vocabulary may restrict their ability to analyze and extract information from the phonetic and semantic components of characters/words. These beginning readers may rely more on rote learning and thus the effect of our working memory training may again be overestimated. In future research older children who have a wider vocabulary should also be tested so that we can compare the effects of metalinguistic and working memory training across age groups. Finally, even if the two training programs have similar effects in typically developing children, it is still possible that one of the two programs has stronger effects in dyslexic children. Thus, it would be worthwhile to replicate the current training study on Chinese dyslexic children. Findings from the dyslexics would be a strong addition to our evaluation of metalinguistic and working memory training in learning to read in Chinese. After all, reading remediation programs are targeted mainly at dyslexic children. Direct evidence that compares the efficacy of training programs on dyslexic children is warranted.

The same arguments about the lack of differences between metalinguistic and working memory trainings also apply to the lack of differences between training in Chinese and English. More powerful studies may still find differences in relative efficacy of the two training methods in the two languages. Also, since our participants are second language learners of English, they may apply their learning strategies in L1 Chinese to their learning of L2 English. This transfer of learning strategies may give rise to similar results between Chinese and English. It is thus imperative to run follow-up studies with native English speaking or more balanced bilingual children to re-examine the effects of different training types on learning to read in English. Moreover, future studies may also find a way to make the working-memory training more engaging to prevent training-specific drop-outs, as was the case in the current study.

In our metalinguistic training that combined phonological and morphological training, both phonological and semantic information of the characters and words were taught. Therefore we were left unsure about whether it was the phonological or morphological or both that drove the improvement on learning to read. In future studies we may have separate groups of phonological and morphological training so that we can disentangle their respective effects.

Another direction for future research is to conduct neural evaluations of the reading training. As the children who received metalinguistic and working memory training showed similar reading gains at the behavioral level, the effects at the neural level may still be different. In the case of dyslexia, similar behavioral reading improvements may result from remediation of deficient processes or from compensatory activities (Gabrieli, 2009). In future training studies, we suggest recording children’s neural activities in response to word reading in Chinese in order to examine training-induced neural plasticity and to determine the mechanisms underlying reading improvements. Moreover, pre-training neural measures may be predictive of training success (Gabrieli et al., 2015; Karipidis et al., 2017), which could offer new perspectives for developing individualized reading training for struggling readers.

## Author Contributions

All authors contributed to the study design. CM, XT, and T-SS contributed test materials. T-SS collected the data. T-SS and UM collaborated on the data analyses and drafted the manuscript. CM provided critical revisions. All authors approved the final version of the manuscript.

## Conflict of Interest Statement

The authors declare that the research was conducted in the absence of any commercial or financial relationships that could be construed as a potential conflict of interest.

## Footnotes

1 Consistency value was estimated by dividing the number of characters which share same phonetic radical AND have the same pronunciation by the total number of characters having the same phonetic radical (Lee et al., 2005).
